# Oral antidiabetic therapy versus early insulinization on glycemic control in newly diagnosed type 2 diabetes patients: a retrospective matched cohort study

**DOI:** 10.1038/s41598-024-66468-1

**Published:** 2024-07-05

**Authors:** Yang-Ming Lee, Pei Ru Lin, Hon-Ke Sia

**Affiliations:** 1 Department of Internal Medicine, Changhua Christian Hospital, Changhua, Taiwan; 2 Big Data Center, Changhua Christian Hospital, Changhua, 500 Taiwan; 3 Graduate Institute of Statistics and Information Science, National Changhua University of Education, Changhua, 500 Taiwan; 4 Department of Endocrinology and Metabolism, Changhua Christian Hospital, 135 Nanhsiao Street, Changhua, 500 Taiwan; 5 Department of Post-Baccalaureate Medicine, College of Medicine, National Chung Hsing University, Taichung, Taiwan

**Keywords:** Endocrinology, Medical research

## Abstract

Our study aims to compare the efficacy of oral antidiabetic therapy to early insulinization on glycemic control among newly diagnosed type 2 diabetes patients in real-world clinical practice. A retrospective cohort study conducted at a medical center in Taiwan analyzed 1256 eligible patients from January 2007 to December 2017. Propensity score matching resulted in well-balanced groups of 94 patients each in the oral antidiabetic drug (OAD) and early insulinization cohorts. Glycemic outcomes were assessed in both groups. Patients exclusively using OAD showed consistently lower glycated hemoglobin (HbA1c) levels at 3, 12, 24, and 36 months compared to insulin users. At later periods, 77.7% of OAD users achieved glycemic control versus 64.9% of insulin users, with a marginally significant difference. Subgroup analyses suggested a trend favoring well-controlled diabetes in the OAD group, though not statistically significant. Our study finds oral antidiabetic therapy is not inferior to early insulinization for glycemic control in newly diagnosed type 2 diabetes patients, irrespective of initial HbA1c levels. This supports oral therapy as a rational treatment option, even in cases with elevated HbA1c at diagnosis.

## Introduction

Diabetes stands as a growing global pandemic characterized by escalating prevalence rates and enduring chronic complications associated with hyperglycemia. Complications include retinopathy, neuropathy, nephropathy, and cardiovascular disease, imposing significant socioeconomic burdens^1–3^. Epidemiologic studies show that type 2 diabetes mellitus has been rising over the past decades, with patients in Taiwan facing a significantly higher risk of mortality compared to the general population^4–6^. The United Kingdom Prospective Diabetes Study (UKPDS) and the Diabetes Control and Complications Trial (DCCT) highlighted "metabolic memory," revealing that early glycemic control in diabetes offers lasting end-organ protection, independent of subsequent treatment or control quality^7–10^. This emphasizes the vital need for timely and optimal glycemic control to prevent diabetes-related complications.

Intensive short-term insulin therapy has been shown to induce glycemic remission in severe, newly diagnosed type 2 diabetes patients^11–13^. Despite the availability of oral antidiabetic drugs (OADs), insulin remains the most potent treatment, providing established benefits including mitigating glucotoxicity, suppressing lipotoxicity, dampening inflammation, abolishing reactive oxygen species (ROS), fostering β-cell recovery, preserving functionality, and bolstering insulin sensitivity^14–25^. However, challenges and barriers associated with insulin therapy, including the risk of hypoglycemia, weight gain, injection anxieties, and logistical inconveniences, may discourage its initiation. Type 2 diabetes is a complex metabolic disorder marked by heightened insulin resistance and diminished β-cell function, exhibiting multifaceted and diverse characteristics^15–17^. It is influenced by a multitude of risk factors, including sedentary lifestyle, dietary habits, obesity, aging, familial predisposition, and ethnic background^1,18–21^. DeFronzo proposed prioritizing the reversal of established pathogenic abnormalities in type 2 diabetes by combining multiple drugs^26^. Since 2000, various OADs targeting diverse mechanisms have been developed. Thus, for newly diagnosed type 2 diabetes patients, OADs alone may be comparable to early insulinization, before the onset of β-cell failure. Our real-world study aims to compare oral antidiabetic therapy versus early insulinization for glycemic control in these patients.

## Methods

### Subjects

**Figure 1:**
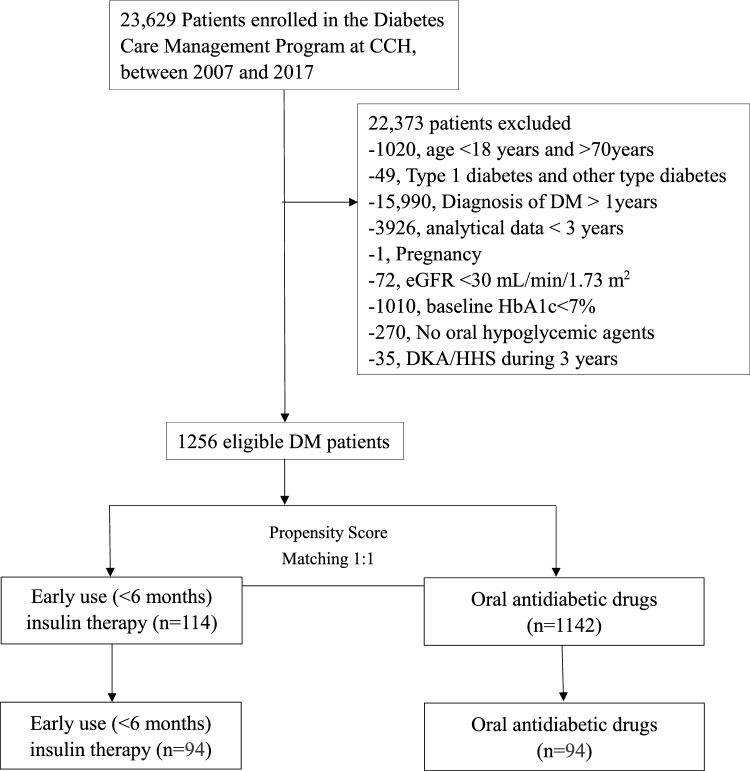
Flow chart of the study population. *CCH* Changhua Christian Hospital, *DM* diabetes mellitus, *eGFR* estimated glomerular filtration rate, *HbA1c* glycated hemoglobin, *DKA* diabetic ketoacidosis, *HHS* hyperglycemic hyperosmolar state.

This retrospective cohort study was conducted at the Changhua Christian Hospital (CCH), Taiwan. A total of 23,629 patients with type 2 diabetes were screened for eligibility using registry data from the Diabetes Case Management Program (DCMP) at the CCH Diabetes Care Center between January 2007 and December 2017. The DCMP provides standardized comprehensive diabetes care including lifestyle assessment, physical examination, laboratory evaluation, and diabetes self-management (DSM) education (such as instruction on nutrition, diet, exercise, medication, self-monitoring of blood glucose (SMBG), and problem-solving skills aimed at reducing related complications). All participants in the program received education during scheduled teaching sessions. Care is delivered by a coordinated multidisciplinary team, including physicians, and certified diabetes educators (registered nurses and dietitians). A detailed description of the program has been reported elsewhere^27^. The diagnosis of T2DM was based on the criteria established by the American Diabetes Association, by satisfying one of the following: a fasting plasma glucose value ≥ 126 mg/dL, a 2-h plasma glucose ≥ 200 mg/dL during a 75-g oral glucose tolerance test, a random plasma glucose ≥ 200 mg/dL in a patient with classic symptoms of hyperglycemia, or a glycated hemoglobin (HbA1c) level of ≥ 6.5%. Excluded from the study were individuals with type 1 and other types of diabetes (n = 49), those with less than 3 years of analyzable data (n = 3926), diabetes duration exceeding 12 months at enrollment (n = 15,990), pregnancy (n = 1), baseline HbA1c < 7 (n = 1010), no anti-diabetic medication treatment (n = 270), DKA/HHS during 3 years (n = 35), age below 18 or above 70 (n = 1020), and an estimated glomerular filtration rate (eGFR) < 30 mL/min/1.73 m^2^ (n = 72). The analysis encompassed 1256 eligible patients, with 1142 in the oral antidiabetic therapy group and 114 in the early insulinization group, following 1:1 propensity score matching to mitigate bias (Fig. 1). Methods were performed in accordance with the relevant guidelines and regulations. The Institutional Review Board of CCH granted the waiver for informed consent and approved the study (IRB No: 210318).

### Data collection

Data were collected from the hospital's electronic medical records, including the DCMP diabetes registry, prescriptions, laboratory data, and CCH research database. Patients with type 2 diabetes were referred to the Diabetes Care Center by diabetes specialists, typically 2 to 6 weeks after their first outpatient clinic visit. Upon enrollment in the DCMP, patients underwent comprehensive assessments, including surveys, physical examinations, and laboratory tests. They received standardized one-on-one diabetes self-management education sessions. A certified diabetes educator evaluated patients' knowledge on glycemic control, willingness towards DSM, frequency of SMBG, and medication adherence through face-to-face interviews post-course^27^.

### Outcome variables

Glycemic control, the primary outcome, was assessed using HbA1c levels measured at baseline and at 3, 6, 9, 12, 24, and 36 months post-enrollment, measured through ion-exchange high-performance liquid chromatography using the VARIANTTM II Turbo system .

### Major exposure variables

Participants who received early insulinization for 3 to 6 months were in the insulin group, while those receiving OADs alone were in the OAD group.

### Variables for subgroup analysis

Participants were categorized based on HbA1c levels (< 9.0% or ≥ 9.0%), gender (male or female), BMI (< 30 kg/m^2^ or ≥ 30 kg/m^2^), and age at onset of diabetes (< 40 or ≥ 40).

### Other control variables

Basic data included age at onset of diabetes, gender, education level, and family history of diabetes. Health-related behaviors included smoking (within the preceding year), alcohol consumption (more than once weekly within the preceding year), and physical activity [regular (≥ 30 min/day, ≥ 3 days/week), occasional (less rigorous than regular exercise), or no exercise]. Knowledge regarding glycemic control was defined as an understanding of the need for and methods of controlling blood glucose. Willingness toward DSM was defined as the motivation to learn self-management techniques. Medication adherence was defined as taking medication regularly at the dose recommended by the physician over the past week. Four-point scales were used to assess the three aforementioned variables. Data were merged into simple dichotomies (i.e., top-two-box vs. bottom-two-box) and categorized as adequate (yes) or inadequate (no) for analysis^27^.

Physical examination included measurement of blood pressure (BP), height, and body weight. Systolic and diastolic BP were measured with the patients in a seated position after a 10-min rest. Body mass index was calculated as body weight (kg)/height (m^2^). Baseline laboratory data included total cholesterol (TC), high-density lipoprotein cholesterol (HDL-C), triglycerides (TG), low-density lipoprotein cholesterol (LDL-C), creatinine, and glutamic pyruvic transaminase (GPT) levels measured using a UniCel DxC 800 Synchron Clinical System (Beckman Coulter, Brea, CA, USA). eGFR was calculated using the equation recommended by the National Kidney Foundation. Data on the major non-psychiatric comorbidities described in the Charlson Comorbidity Index (CCI) during the year preceding enrolment were collected from the CCH research database. Major comorbidities, including congestive heart failure (CHF), coronary artery disease (CAD), cerebrovascular accident (CVA), hypertension (HTN), hyperlipidemia, chronic kidney disease (CKD), cancer, cirrhosis, rheumatoid arthritis (RA), myocardial infarction (MI), peripheral vascular disease (PVD) and dysrhythmia were analyzed as independent variables^27^.

### Statistical analysis

Frequency counts with percentages and mean ± standard deviation (SD) were used to describe categorical and continuous variables, respectively. Differences between the two groups were assessed using the chi-square test for categorical variables and the Student’s t-test for continuous variables. To mitigate the influence of confounding variables, we employed 1:1 propensity score matching. Kaplan–Meier method was utilized to generate cumulative incidence curves for well-controlled events, and discrepancies between these curves were assessed using log-rank tests. Cox proportional hazard models were employed to evaluate the relationship between insulin use status and well-controlled events. The multivariate Cox proportional regression model was adjusted for the propensity score. Hazard ratios (HR) with 95% confidence intervals (CI) were used to express the results. Subgroup analyses were conducted to evaluate the association between insulin and glycemic control in subgroups stratified by various HbA1c levels (≥ 9.0% or < 9.0%), gender (male or female), BMI (≥ 30 kg/m^2^ or < 30 kg/m^2^), and age at onset of diabetes (≥ 40 or < 40). All analyses were two-tailed and performed using IBM SPSS Statistics version 22 (IBM Corp., Armonk, NY, USA) and SAS software, version 9.4 (SAS Institute Inc., Cary, NC, USA). The significance level was set at 0.05.

### Informed consent

This study was approved after a full ethical review by the Institutional Review Board (IRB) of the Changhua Christian Hospital (approval number 210318), and the IRB waived the need for consent. Those data were accessed anonymously.

## Results

### Characteristics of patients

**Figure 2:**
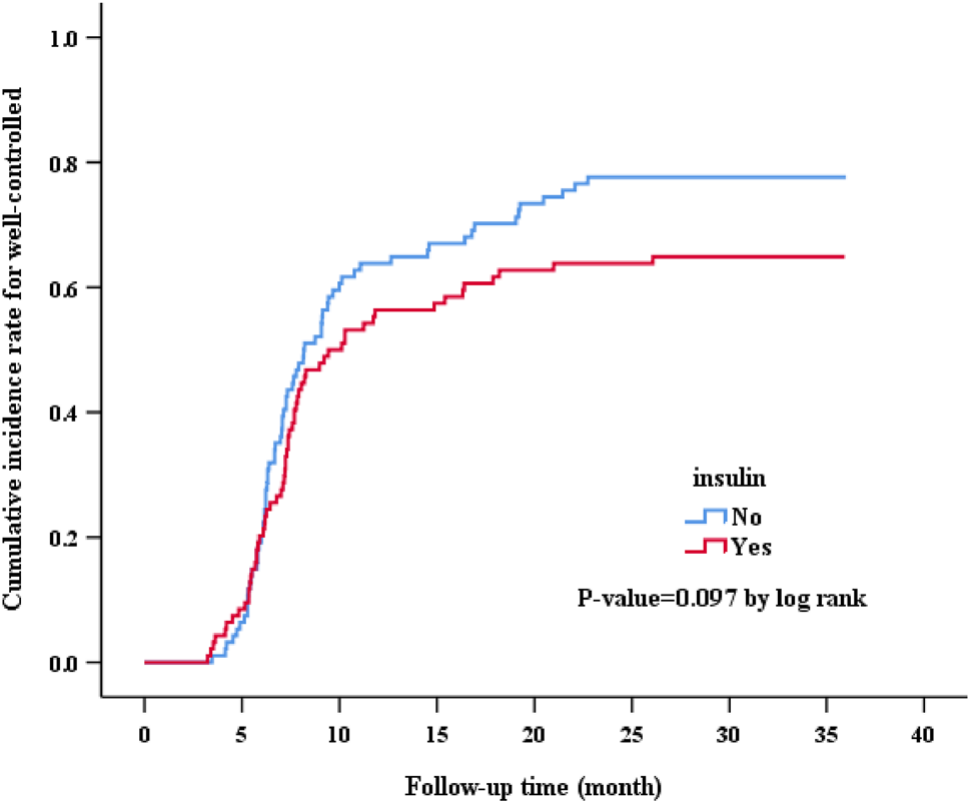
KM plot. Illustrating that the cumulative incidence of well-controlled diabetes between the OAD and early insulinization groups.

Out of the 23,629 patients screened, 1256 met the eligibility criteria. Following propensity score matching (1:1), each group consisted of 94 patients. The OAD group had an average age of 51.96 ± 11.03 years and an average BMI of 26.74 ± 4.6 kg/m^2^, while the insulin group had an average age of 49.71 ± 11.37 years and an average BMI of 26.34 ± 5.12 kg/m^2^ (Table 1). Both groups shared similar demographic traits, encompassing age, gender, education level, family history of diabetes, smoking and alcohol consumption, physical activity, knowledge of glycemic control, willingness towards DSM, medication adherence, BMI, mean BP, total cholesterol, triglycerides, HDL, LDL, eGFR, GPT, comorbidities at baseline (such as CHF, CVA, CAD, HTN, hyperlipidemia, CKD, cancer, cirrhosis, RA, MI, PVD, and dysrhythmia) (Table 1). The baseline HbA1c levels were similar between the OAD and early insulinization groups (HbA1C 10.73 ± 2.54 vs. 10.92 ± 2.05, p = 0.565) (Table 2). However, over the course of 36 months, the OAD consistently demonstrated lower HbA1c levels at 3, 12, 24, and 36 months compared to the early insulinization group. (HbA1C: 7.66 ± 1.94 vs. 8.13 ± 1.91, p = 0.095; 7.07 ± 1.46 vs. 7.55 ± 1.94, p = 0.061; 7.01 ± 1.19 vs. 7.49 ± 1.58, p = 0.018; 7.03 ± 1.05 vs. 7.5 ± 1.66, p = 0.023, respectively) (Table 2). After the index period, 77.7% in the OAD group and 64.9% of patients in the early insulinization group achieved glycemic control, with a marginally significant difference (p = 0.053) (Table 2). Time to control did not significantly differ between the OAD group (14.42 ± 11.17 months) and the early insulinization group (17.54 ± 13 months) (p = 0.080) (Table 2). The OAD group exhibited a higher incidence of well-controlled diabetes than the insulin group, but the difference was not statistically significant (p = 0.097, log-rank test) (Fig. 2).

**Table 1:** Baseline demographic comparisons: analyzing characteristics, variables, and comorbidities in subjects with oral antidiabetic therapy versus early insulinization.

	Oral antidiabetic therapy	Early insulinization	P-value
Sample size	94	94	
Age	51.96 ± 11.03	49.71 ± 11.37	0.171
Gender, male	45 (47.9%)	58 (61.7%)	0.057
Level of education: no	3 (3.2%)	5 (5.3%)	0.865
Primary school	18 (19.1%)	16 (17.0%)	
Secondary or high school	46 (48.9%)	44 (46.8%)	
University or above	27 (28.7%)	29 (30.9%)	
Family history of DM: Yes	64 (68.1%)	65 (69.1%)	0.875
Current smoking	12 (12.8%)	19 (20.2%)	0.169
Alcohol drinking	4 (4.3%)	3 (3.2%)	0.700
Physical activity: No exercise	44 (46.8%)	44 (46.8%)	0.835
Occasional exercise	22 (23.4%)	25 (26.6%)	
Regular exercise	28 (29.8%)	25 (26.6%)	
Knowledge regarding GC: yes	44 (46.8%)	50 (53.2%)	0.381
Willingness toward DSM: yes	89 (94.7%)	84 (89.4%)	0.178
Perform SMBG: yes	93 (98.9%)	94 (100%)	0.316
Medication adherence: Yes	92 (97.9%)	91 (96.8%)	0.650
Insulin secretagogues	51 (54.3%)	38 (40.4%)	0.058
Non-insulin secretagogues	73 (77.7%)	82 (87.2%)	0.084
*Clinical variables*			
BMI (kg/m^2^)	26.74 ± 4.6	26.34 ± 5.12	0.581
Mean BP (mmHg)	94.66 ± 9.61	96.79 ± 11.25	0.164
Total cholesterol (mg/dL)	176.76 ± 42.06	176.43 ± 41.56	0.957
Triglycerides (mg/dL)	157.06 ± 156.2	134.71 ± 82.21	0.222
HDL (mg/dL)	46.7 ± 11.41	45.8 ± 13.02	0.613
LDL (mg/dL)	103.81 ± 34.32	105.55 ± 35.56	0.733
eGFR (mL/min/1.73m^2^)	100.85 ± 28.51	105.94 ± 43.19	0.342
GPT	31.44 ± 26.78	39.05 ± 42.92	0.146
*Comorbidity at baseline*			
CHF	8 (8.5%)	9 (9.6%)	0.799
CVA	0 (0%)	3 (3.2%)	0.246
CAD	1 (1.1%)	3 (3.2%)	0.621
HTN	44 (46.8%)	47 (50.0%)	0.662
Hyperlipidemia	63 (67.0%)	53 (56.4%)	0.134
CKD	3 (3.2%)	1 (1.1%)	0.621
Cancer	2 (2.1%)	2 (2.1%)	1.000
Cirrhosis	4 (4.3%)	2 (2.1%)	0.682
RA	1 (1.1%)	2 (2.1%)	1.000
MI	0 (0%)	0 (0%)	–
PVD	1 (1.1%)	1 (1.1%)	1.000
Dysrhythmia	0 (0%)	0 (0%)	–

Results are expressed as mean ± SD or n (%).

*DM* diabetes mellitus, *GC* glycemic control, *DSM* diabetes self-management, *SMBG* self-monitoring of blood glucose, *HbA1c* hemoglobin A1c, *BMI* body mass index, *BP* blood pressure, *HDL-C* high-density lipoprotein cholesterol, *LDL-C* low-density lipoprotein cholesterol, *eGFR* estimated glomerular filtration rate, *GPT* glutamic pyruvic transaminase, *CHF* congestive heart failure, *CAD* coronary artery disease, *CVA* cerebrovascular accident, *CKD* chronic kidney disease, *PVD* peripheral vascular disease.

**Table 2:** Oral antidiabetic therapy versus early insulinization on glycemic control after propensity score matching.

	Oral antidiabetic therapy	Early insulinization	P-value
HbA1C at baseline (%)	10.73 ± 2.54	10.92 ± 2.05	0.565
HbA1C at 3-month (%)	7.66 ± 1.94	8.13 ± 1.91	0.095
HbA1C at 6-month (%)	6.87 ± 1.13	7.43 ± 1.86	0.013
HbA1C at 9-month (%)	6.94 ± 1.36	7.52 ± 1.89	0.017
HbA1C at 12-month (%)	7.07 ± 1.46	7.55 ± 1.94	0.061
HbA1C at 18-month (%)	7.06 ± 1.34	7.58 ± 1.72	0.022
HbA1C at 24-month (%)	7.01 ± 1.19	7.49 ± 1.58	0.018
HbA1C at 30-month (%)	7.14 ± 1.24	7.53 ± 1.4	0.044
HbA1C at 36-month (%)	7.03 ± 1.05	7.5 ± 1.66	0.023
*Outcome after index date*			0.023
Well-controlled	73 (77.7%)	61 (64.9%)	0.023
*Follow-up time*			0.023
Time to well-controlled (month)	14.42 ± 11.17	17.54 ± 13	0.023

Results are expressed as mean ± SD or n (%).

*HbA1c* hemoglobin A1c. Index date: the date of enrollment. Well-controlled: HbA1c < 7.

Subgroup analyses revealed consistently higher rates of well-controlled diabetes in the OAD group compared to the early insulinization group across baseline HbA1c levels (< 9.0%: aHR:3.38, p = 0.005; ≥ 9.0%: aHR: 1.09, p = 0.662), gender (males: aHR: 1.90, p = 0.005; females: aHR: 1.19, p = 0.551), age(< 40: aHR:1.33, p = 0.441; ≥ 40: aHR: 1.36, p = 0.121),and BMI (< 30 kg/m^2^: aHR: 1.26, p = 0.226; ≥ 30 kg/m^2^: aHR: 1.65, p = 0.239) (Table 3). However, these differences did not reach statistical significance. (Table 3).

**Table 3:** Subgroup analysis of well-controlled type 2 diabetes in oral antidiabetic therapy versus early insulinization.

Subgroup	n	cHR (95% CI)	P-value	aHR* (95% CI)	P-value
*HbA1c*					
< 9%	44	2.11 (0.99, 4.49)	0.054	2.10 (0.99, 4.48)	0.055
≥ 9%	182	1.11 (0.80, 1.56)	0.530	1.12 (0.80, 1.57)	0.496
*Gender*					
Male	145	1.15 (0.79, 1.67)	0.460	1.16 (0.80, 1.69)	0.425
Female	81	1.53 (0.89, 2.63)	0.127	1.53 (0.89, 2.63)	0.128
*Age*					
< 40	55	0.93 (0.51, 1.71)	0.814	0.93 (0.50, 1.72)	0.817
≥ 40	171	1.42 (0.99, 2.02)	0.057	1.40 (0.98, 2.00)	0.066
*BMI*					
< 30	186	1.30 (0.93, 1.81)	0.124	1.29 (0.93, 1.80)	0.133
≥ 30	40	1.19 (0.54, 2.61)	0.666	1.51 (0.65, 3.50)	0.339

*Adjusted for propensity score.

**Figure 3:**
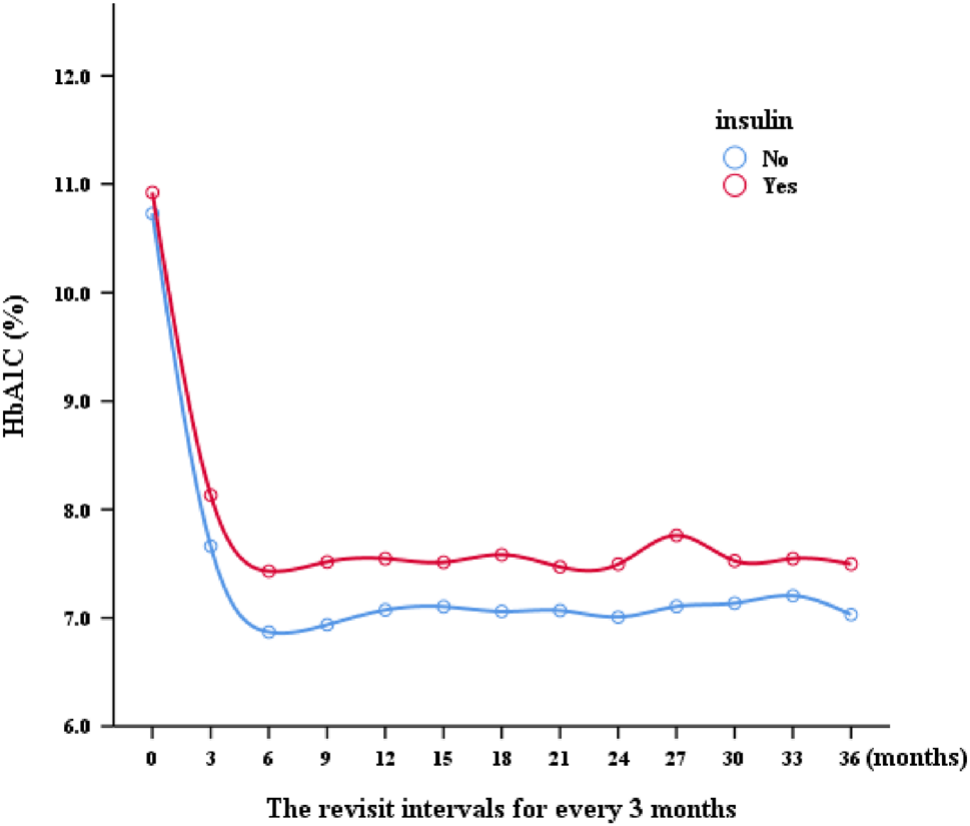
Comparison of mean HbA1C levels at different visit between the OAD and early insulinization groups.

In general, irrespective of glycemic control (HbA1c < 9.0% or ≥ 9.0%), gender (male or female), diabetes onset age (age < 40 or ≥ 40), and BMI (BMI < 30 kg/m^2^ or ≥ 30 kg/m^2^), the OAD group exhibited a more substantial reduction in HbA1c levels compared to the insulin group (Fig. 3).

## Discussion

We found that oral antidiabetic therapy was not inferior to early insulinization on glycemic control in newly diagnosed type 2 diabetes patients, irrespective of baseline HbA1c, BMI, gender and age. While the initial baseline HbA1c levels of our study participants were notably elevated (ranging from 10.75 to 10.92%), the group receiving oral antidiabetic therapy demonstrated more significant reductions in HbA1c levels over the course of 3, 12, 24, and 36 months. This led to a higher rates of well-controlled glycemic levels (77.7% vs. 64.9%), and a shorter timeframe (14.42 ± 11.17 vs. 17.54 ± 13 months) in OAD group compared to early insulinization group.

Our study findings differ from prior investigations conducted by Weng et al., Ryan et al., and Karacaer et al.^11,12,28^, which showed a greater percentage of patients attaining target glycemic control within a shorter timeframe in the insulin group compared to those receiving OADs. Their methods involved continuous subcutaneous infusion insulin (CS II) or multiple daily insulin injections (MDI) with daily dosage adjustments^11,12,28^, contrasting our insulin approach characterized by reduced intensity of injection and monitoring. In newly diagnosed type 2 diabetes patients, early insulinization is beneficial with high-intensity titration and monitoring, otherwise, oral antidiabetic therapy may be a suitable alternative.

In the past two decades, oral antidiabetic drugs (OADs) have undergone significant advancements. DeFronzo's 'ominous octet' delineates eight factors contributing to the pathophysiology of type 2 diabetes, advocating combination therapy involving diet, exercise, metformin, thiazolidinediones (TZD), and glucagon-like peptide-1 (GLP-1) receptor agonists. This approach aims to enhance insulin sensitivity, preserve β-cell function, and facilitate weight loss in individuals with type 2 diabetes^26^. Inadequate dose adjustment and monitoring of insulin therapy may increase the risk of hypoglycemia and weight gain compared to OADs alone, potentially undermining insulin's benefits. Although both groups in our study demonstrated similar rates of SMBG (98.9% vs. 100%), this measure alone may not fully capture the intensity and frequency of SMBG practices. Variations in self-monitoring of blood glucose (SMBG) patterns have a notable impact on physicians' decisions regarding medication regimens, particularly within the insulin-treated group^29^. Such variations have the potential to worsen clinician inertia. For individuals newly diagnosed with type 2 diabetes, it is essential to prioritize addressing the underlying pathophysiological defect by implementing suitable medications, lifestyle adjustments, and self-management strategies. This comprehensive approach should be prioritized over relying solely on early insulinization.

The potential for hypoglycemia poses a significant concern with insulin injections. Attending physicians may have been cautious about administering intensive insulin treatment due to this fear. Similarly, insulin users themselves may have experienced anxiety about hypoglycemia, leading to irregular treatment or self-adjustment of insulin dosages. Finally, The UKPDS did not support a benefit of insulin treatment on macrovascular complications^30^ but metformin may provide such a benefit in overweight patients^31^. Insulin use has been found to be associated with a significantly higher risk of cancer^32^, and the mortality from breast cancer among female patients with T2D is significantly higher if insulin has been used for more than 3 years^33^. According to prospective follow-up studies conducted in Taiwan, insulin use together with smoking may significantly increase the risk of mortality from hepatocellular carcinoma, bladder cancer, and pancreatic cancer in patients with T2D, among others^34–36^.

Subgroup analysis revealed a significantly higher rate of well-controlled cases among those with a baseline HbA1c < 9.0% using OADs alone (HR: 3.38, p = 0.005) compared to those undergoing early insulinization. Although not achieving statistical significance, this implies that OADs alone might be more favorable for individuals with lower baseline HbA1c levels. This observation aligns with current guidelines^37^.

Our study's strength lies in its utilization of longitudinal data, which establishes a temporal relationship between medical therapy and glycemic control. This approach mitigates the potential for reverse causality, a limitation often encountered in cross-sectional studies. This study has notable limitations that warrant acknowledgment. First, one major limitation is the lack of a randomized clinical trial design. Despite using propensity score matching to address selection bias, it may not fully suffice. The decision to use insulin is influenced by physicians' attitudes, educators' persuasion, and patient acceptance, potentially introducing selection bias in comparing insulin to oral antidiabetic agents. Second, the extensive time span of patient enrollment from 2007 to 2017 has resulted in diverse insulin regimens and variations in oral antidiabetic agents, which may ultimately underestimate our study outcomes. Third, the study's capacity to establish a clear causal relationship is restricted due to the absence of randomization. However, the utilization of propensity score matching has mitigated the potential for selection bias and addressed the effects of confounders. Fourth, as a retrospective study, the causal interpretation of this study was limited. Fifth, the potential impact of certain unmeasured confounding factors on the observed association cannot be definitively excluded. For instance, the precise effects of dietary habits, actual intensity of physical activity, intensity of self-monitoring of blood glucose (SMBG), frequency and severity of hypoglycemia, and changes in body weight on our study population remain unclear.

Lastly, although this study may provide some real-world information in a specific hospital, its findings might be biased because of some major limitations. Therefore, whether the findings can be generalized to other areas in Taiwan or other countries remains to be investigated.

## Conclusions

Early insulinization may not significantly enhance glycemic control in newly diagnosed type 2 diabetes patients compared to utilizing OADs alone. Simply initiating insulin early is insufficient; attaining optimal control necessitates a structured insulin titration protocol and diligent self-monitoring of blood glucose. Our real-world data support considering oral antidiabetic therapy as a reasonable treatment option for newly diagnosed type 2 diabetes patients, even those with elevated baseline HbA1c levels (Supplementary Table, Supplementary Figure).

### Supplementary Information

Supplementary Tables.

Supplementary Figures.

## Data Availability

The dataset utilized in the study is not accessible due to confidentiality regulations outlined in the Personal Information Protection Act enforced by the Taiwanese Government since 2012. For additional details regarding data acquisition, interested parties may contact the primary author upon request.
