# Supplementary material for: Oral antidiabetic therapy versus early insulinization on glycemic control in newly diagnosed type 2 diabetes patients: a retrospective matched cohort study
Source: Sci Rep. 2024 Jul 5;14:15491. doi: 10.1038/s41598-024-66468-1 (PMC11226661; doi:10.1038/s41598-024-66468-1)
Supplement: Supplementary file 1 — Supplementary Tables. [file 41598_2024_66468_MOESM1_ESM.docx]

S1. Effects of early use (<6 months) insulin therapy on well controlled with type 2 diabetes.

|  | **Well-controlled** | | | |
| --- | --- | --- | --- | --- |
|  | **cHR (95% CI)** | **P-value** | **aHR (95% CI)** | **P-value** |
| Oral antidiabetic therapy | 1.33(0.95,1.87) | 0.098 | 1.33(0.94,1.86) | 0.106 |

Adjusted for propensity score.
