# Supplementary material for: Oral antidiabetic therapy versus early insulinization on glycemic control in newly diagnosed type 2 diabetes patients: a retrospective matched cohort study
Source: Sci Rep. 2024 Jul 5;14:15491. doi: 10.1038/s41598-024-66468-1 (PMC11226661; doi:10.1038/s41598-024-66468-1)

S2. Subgroup Analysis: Samples categorized by baseline HbA1C, gender, age, and BMI.

**a. HbA1C <9% b. HbA1C** ≥**9%**


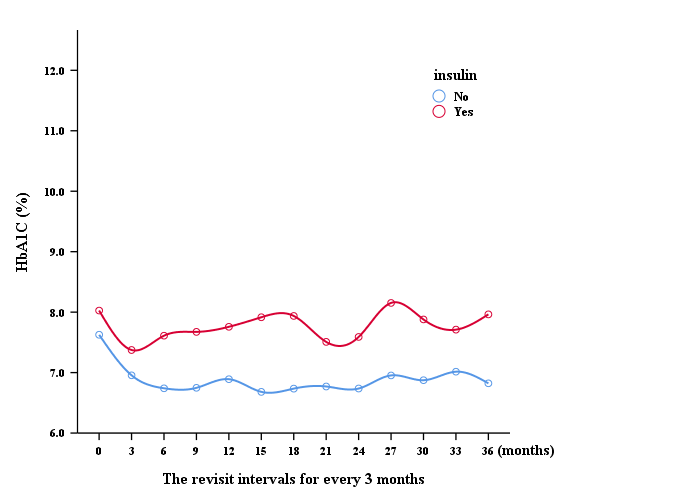

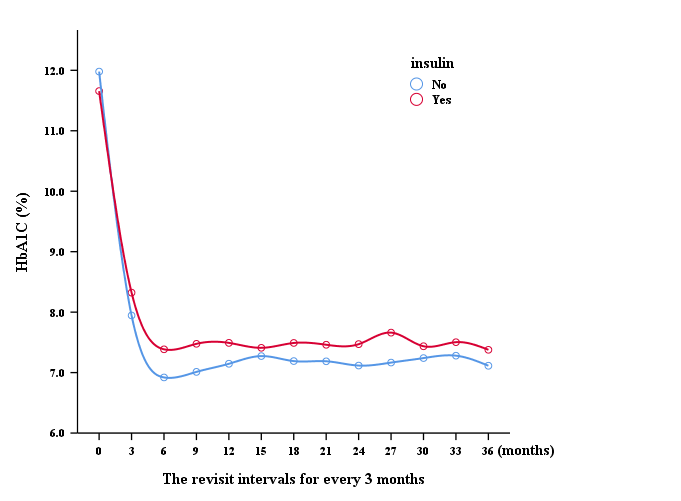


**c. Male d. Female**


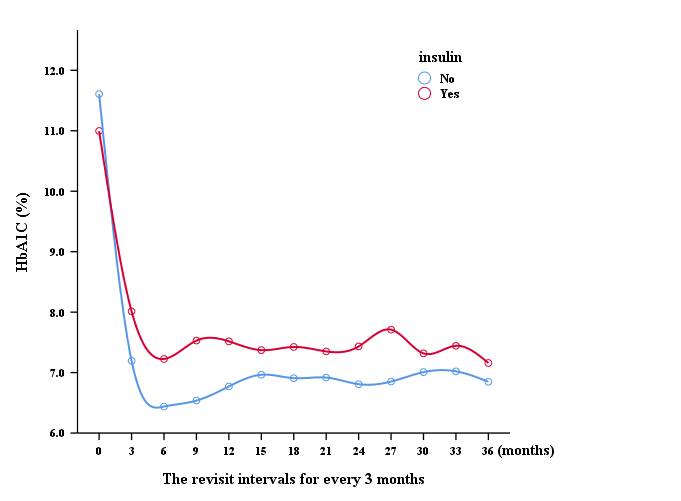

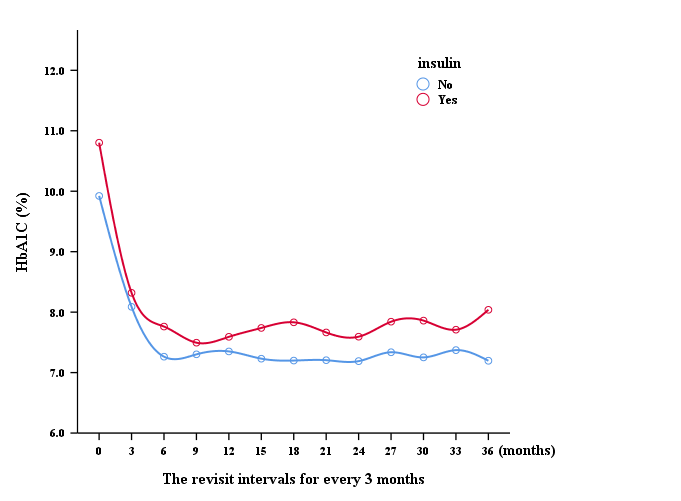


**e. Age <40 f. Age ≥40**


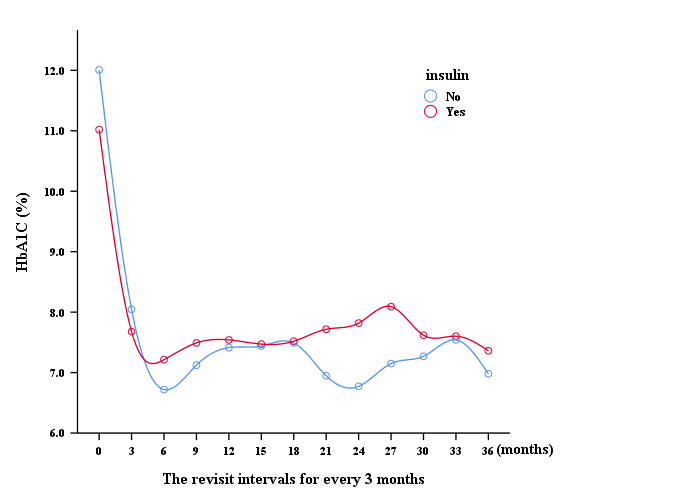

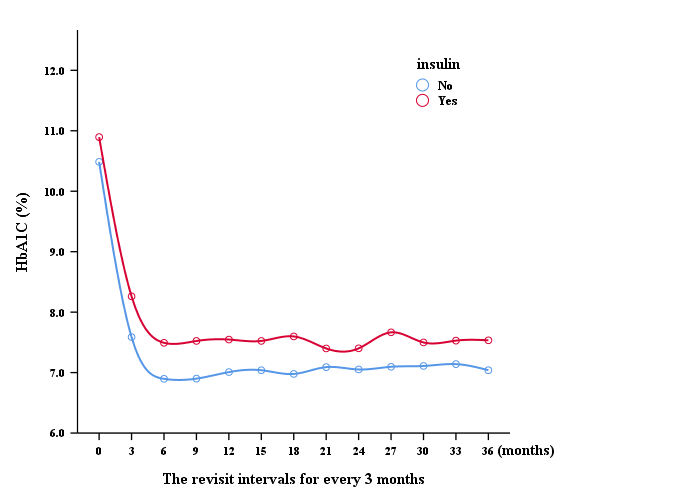


**g. BMI <30 h. BMI ≥30**


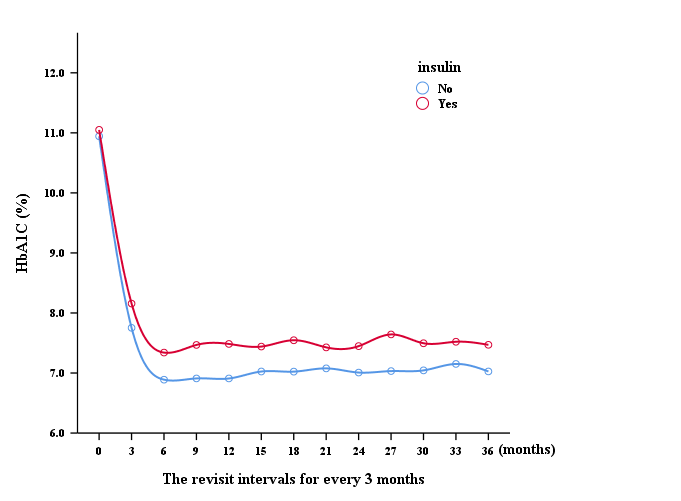

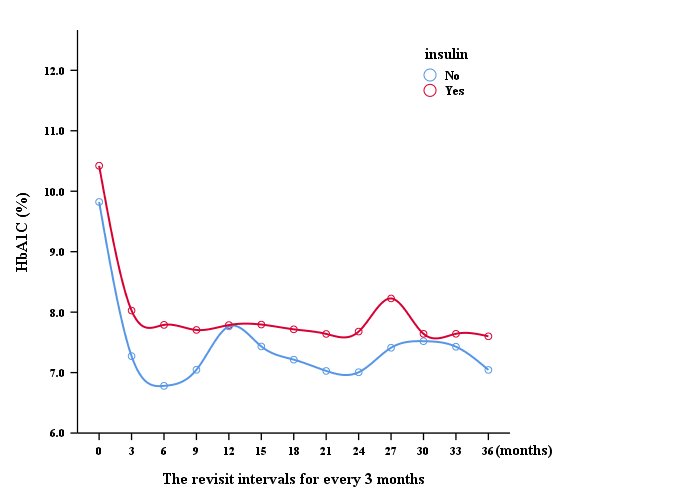

Supplement: Supplementary file 2 — Supplementary Figures. [file 41598_2024_66468_MOESM2_ESM.docx]
